# Impact of hypertensive disorders of pregnancy on neonatal outcomes among infants born at 24^+0^–31^+6^ weeks’ gestation in China: A multicenter cohort study

**DOI:** 10.3389/fped.2023.1005383

**Published:** 2023-02-23

**Authors:** Jianfang Ge, Xinyue Gu, Shanyu Jiang, Ling Yang, Xiaoyan Li, Siyuan Jiang, Beibei Jia, Caihua Chen, Yun Cao, Shoo Lee, Xiaopeng Zhao, Yong Ji, Wenhao Zhou

**Affiliations:** ^1^ Department of Neonatology, Shanxi Provincial Children's Hospital, Taiyuan, China; ^2^ NHC Key Laboratory of Neonatal Diseases, Children's Hospital, Fudan University, Shanghai, China; ^3^ Department of Neonatology, Wuxi Maternity and Child Health Care Hospital, Wuxi, China; ^4^ Department of Neonatology, Hainan Women and Children's Medical Center, Haikou, China; ^5^ Department of Paediatric, University of Toronto, Toronto, ON, Canada; ^6^ Department of Neonatology, Guangzhou Women and Children's Hospital, Guangzhou, China

**Keywords:** hypertensive disorder of pregnancy, very preterm infants, neonatal outcomes, preeclampsia/eclampsia, neonatal mortality and morbidity

## Abstract

**Objective:**

To describe the rate of hypertensive disorder of pregnancy (HDP) among mothers of very preterm infants (VPIs) admitted to Chinese neonatal intensive care units (NICUs), and to investigate the relationship between HDP and the outcomes of VPIs.

**Study design:**

Cohort study of all VPIs born at a gestational age of 24^+0^–31^+6^ weeks and admitted to 57 tertiary NICUs of the Chinese Neonatal Network (CHNN) in 2019. Infants with severe congenital anomalies or missing maternal HDP information were excluded. Two multivariate logistic regression models were generated to assess the relationship between HDP and neonatal outcomes.

**Results:**

Among 9,262 infants enrolled, 1,744 (18.8%) infants were born to mothers with HDP, with an increasing incidence with increasing gestational age. VPIs born to mothers with HDP had higher gestational age but lower birth weight and were more likely to be small for gestational age. Mothers with HDP were more likely to receive antenatal steroids, MgSO_4_ and cesarean section. Infants in the HDP group showed higher observed rates of mortality or any morbidity than infants in the non-HDP group (50.2% vs. 47.2%, crude odds ratio (OR) 1.13, 95% CI 1.02–1.26). However, the associations between HDP and adverse outcomes were not significant after adjustment. In the HDP group, mothers of 1,324/1,688 (78.4%) infants were diagnosed with preeclampsia/eclampsia. Infants born to mothers with preeclampsia/eclampsia had significantly lower odds of early death and severe retinopathy of prematurity.

**Conclusions:**

Nearly one-fifth of VPIs were born to mothers with HDP in Chinese NICUs. No significant association was identified between HDP and adverse neonatal short-term outcomes of VPIs, while long-term follow-up of these infants is needed.

## Introduction

Hypertensive disorder of pregnancy (HDP) is one of the most common complications during pregnancy (1, 2). HDP occurs in approximately 10% of all pregnancies (3), and the incidence of HDP has been increasing over the past few decades (3, 4). In recent years, the research on the relationship between HDP and adverse pregnancy outcomes (APOs) of offspring has gradually increased from fetus to adulthood (5), involving the nervous system (6), cardiovascular system (7), immune system (8), respiratory system (9), emotional behavior system (10), etc.

HDP is a significant cause of mortality and morbidities for both mothers and neonates (11, 12). As a determined risk factor for preterm birth, HDP may have an extended adverse impact on preterm infants. Very preterm infants (VPIs,<32 weeks) accounted for 16% of preterm infants with the highest mortality and morbidities (13). Several studies investigated the impact of HDP on the outcomes of preterm infants and yielded inconsistent results. Some studies reported reduced risks of mortality (14), intraventricular hemorrhage (IVH) (14–16), and periventricular leukomalacia (PVL) (15) but increased risks of retinopathy of prematurity (ROP) (17) and necrotizing enterocolitis (NEC) (18) associated with HDP, whereas others demonstrated that preterm infants of mothers with HDP were very similar to preterm infants of mothers without HDP on most outcomes (19–21).

The incidence of HDP in China has been reported to be higher than in other areas of the world (22). However, very limited studies have reported the impact of HDP on the outcomes of VPI in the Chinese population. One recent regional study showed a high rate (24.4%) of HDP for mothers of VPIs in a Chinese province. They also found an increased risk of bronchopulmonary dysplasia (BPD) and a decreased risk of severe IVH among infants born to mothers with HDP (23). Nonetheless, there is a lack of national data currently. Thus, our study used the largest cohort of VPIs in China from the Chinese Neonatal Network, aiming to demonstrate the rate of HDP among mothers of VPIs and to investigate the association between HDP and neonatal outcomes. We also further assessed the impact of preeclampsia/eclampsia on neonatal outcomes among VPIs born to HDP mothers.

## Methods

### Data source

The Chinese Neonatal Network (CHNN) is a national network of Chinese tertiary neonatal intensive care units (NICUs) with the primary goal to conduct high-quality collaborative research dedicated to the improvement of neonatal-perinatal health in China. Hospitals enrolled in the CHNN are required to be tertiary referral hospitals with large neonatal services with recognized expertise in caring for high-risk neonates. CHNN has established and maintained a standardized clinical database of preterm infants at <32 weeks’ gestation or <1500 g in participating NICUs throughout China to monitor outcomes and care practices from January 1, 2019. A total of 57 hospitals from 25 provinces throughout China collected whole-year data using the CHNN database in 2019. These 57 hospitals include 3 national children’s medical centers, 4 regional children’s medical centers, and 30 provincial perinatal or children’s medical centers. The other 19 hospitals comprised major referral centers in large cities across China. Forty-three hospitals were perinatal centers with birthing facilities, and 14 were free-standing children’s hospitals that only admitted outborn infants. All hospitals had the ability to provide complicated care for infants at <32 weeks’ gestation.

### Study population

Infants admitted to CHNN participating hospitals from January 1 to December 31, 2019, with gestational age at 24^+0^–31^+6^ weeks were included in this study. Stillborn, delivery room death, and infants transferred to nonparticipating hospitals within 24 h after birth were not captured by the database. Readmissions and transfers between participating hospitals were tracked as data from the same infants. Infants were followed until NICU discharge, transfer, or death. Infants who had severe congenital anomalies and missing maternal HDP information were excluded. This study was approved by the Ethics Review Board of Children’s Hospital of Fudan University (2018–296), which was recognized by all participating hospitals. Waiver of consent was granted at all sites.

### Data collection

Trained data abstractors were responsible for data acquisition in each hospital. Data were directly entered into a customized database with built-in error checking and a standard manual of operations and definitions. Data were electronically transmitted to the CHNN coordinating center at Children’s Hospital of Fudan University with patient identity kept confidential. Site investigators were responsible for data quality control at each site.

### Exposure

HDP in our database was diagnosed according to the 2015 Chinese Guideline on Hypertensive Disorder of Pregnancy, defined as systolic blood pressure ≥140 mmHg and/or diastolic blood pressure ≥90 mmHg either at the beginning of pregnancy (chronic hypertension) or after 20 weeks’ gestation (gestational hypertension) (24). HDP with or without other pregnancy complications were all included in the exposure group. Preeclampsia/eclampsia data are available within HDP mothers, based on the obstetric record of whether preeclampsia or eclampsia is present or not. Preeclampsia is characterized by new-onset maternal hypertension and proteinuria at or after 20 weeks of gestation. Eclampsia is one or more convulsions in association with the syndrome of preeclampsia (25).

### Outcomes

The primary outcome of this study was mortality or any major morbidity. Major morbidities included sepsis, NEC stage II or above, severe ROP, severe brain injury, and BPD. Sepsis was defined as positive blood or cerebrospinal fluid culture, while early- or late-onset sepsis were defined as sepsis that occurred within or after 72 h after birth. NEC was defined according to Bell’s criteria (26). Severe ROP was diagnosed as stage ≥3 according to the International Classification of ROP (27). Severe brain injury included IVH ≥grade 3 according to Papile’s criteria (28) and cystic PVL. BPD was defined as ventilation or oxygen dependency at 36 weeks’ postmenstrual age or at discharge/transfer/death before 36 weeks (29).

### Covariates definitions

Gestational age was determined using the hierarchy of best obstetric estimates based on prenatal ultrasound, menstrual history, obstetric examination, or all three. If the obstetric estimate was not available or was different from the postnatal estimate of gestation by more than 2 weeks, the gestational age was estimated using the Ballard Score (30). Small for gestational age (SGA) was defined as birth weight <10th percentile for the gestational age according to the Chinese neonatal birth weight values, while large for gestational age (LGA) was defined as birth weight <10th percentile (31). Prenatal care was defined as ≥1 pregnancy-related hospital visit during pregnancy. Transport Risk Index of Physiologic Stability (TRIPS) score was used as an illness severity score on NICU admission (32).

### Statistical analysis

Descriptive analysis was applied to demonstrate the rate of HDP among mothers of VPIs in China. Demographic characteristics for mothers and infants were compared between infants born to mothers with and without HDP. Categorical variables were tested by Pearson chi-square test, and continuous variables were tested by Student’s *t*-test or Wilcoxon rank sum test as appropriate.

To investigate the association between HDP and neonatal outcomes, two multivariate logistic regression models were generated, based on Generalized Estimating Equations (GEE) accounting for cluster effect within each site. In model 1, adjustment was made for maternal age, primigravida, maternal diabetes, gestational age, birth weight, sex, and multiple births, while model 2 further adjusted for prenatal care, antenatal corticosteroids, cesarean section, MgSO_4_, and inborn/outborn status on the basis of model 1. Subgroup analysis was done by gestational age strata of 24^+0^–27^+6^ weeks and 28^+0^–31^+6^ weeks.

To further investigate the association between preeclampsia and neonatal outcomes, the same analysis plan was applied to compare infants born to mothers with no HDP, HDP without preeclampsia/eclampsia, and HDP with preeclampsia/eclampsia.

The data management and all statistical analyses were performed using SAS version 9.4 (SAS Institute, Inc., Cary, NC, United States). A two-sided *P* value of 0.05 was used to determine statistical significance.

## Results

### Study population and rate of HDP

**Figure 1:**
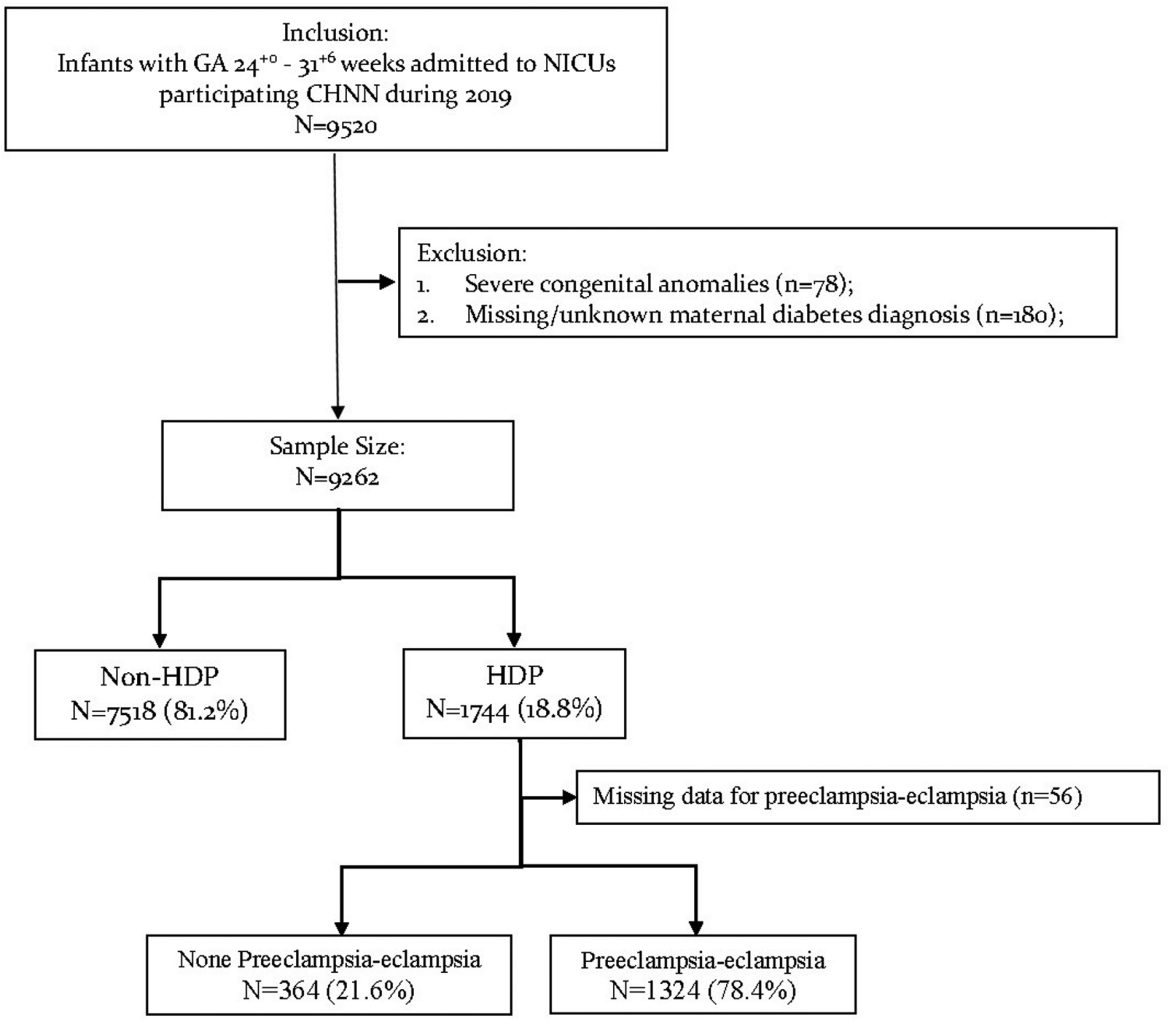
Data flow of study population.

Of the 9,520 neonates admitted to CHNN hospitals with gestational age at 24^+0^–31^+6^ weeks in 2019, 78 infants were excluded for severe congenital anomalies and 180 infants were excluded for missing maternal HDP information (Figure 1). The remaining 9,262 VPIs were finally included in our study.

Among all infants enrolled, 1,744/9,262 (18.8%) infants were born to mothers with HDP while 7,518/9,262 (81.2%) infants were born to non-HDP mothers (Table 1). The rates of maternal HDP increased significantly with increasing gestational age (Table 1, tread test *P* value <0.01). Among infants at 24^+0^–27^+6^ weeks’ gestation, 158/1,567 (10.1%) were born to mothers with HDP. While among infants at 28^+0^–31^+6^ weeks, 1,586/7,695 (20.6%) were born to mothers with HDP.

**Table 1:** Maternal hypertensive disorders of pregnancy for very preterm infants in China.

Gestational age (weeks)	Total number of infants	HDP, *n* (%)^a^	Preeclampsia/eclampsia, *n* (%)^b^
24	91	7 (7.7)	3/6 (50.0)
25	221	10 (4.5)	5/10 (50.0)
26	443	40 (9.0)	26/35 (74.3)
27	812	101 (12.4)	71/98 (72.4)
28	1,377	222 (16.1)	170/214 (79.4)
29	1,731	323 (18.7)	240/311 (77.2)
30	2,115	460 (21.7)	356/448 (79.5)
31	2,472	581 (23.5)	453/566 (80.0)
Total	9,262	1,744 (18.8)	1,324/1,688 (78.4)

HDP, hypertensive disorder of pregnancy.

^a^ Cochran–Armitage trend test p < .01.

^b^ 56 cases, whose mother had HDP, have missing data on preeclampsia/eclampsia.

Among the HDP group, mothers of 1,324/1,688 (78.4%) infants were diagnosed with preeclampsia/eclampsia. The proportion of preeclampsia/eclampsia among HDP mothers also increased with increasing gestational age (tread test *P* value <0.01). Among infants at 24^+0^–27^+6^ weeks’ gestation, mothers of 105/149 (70.5%) neonates were diagnosed with preeclampsia/eclampsia. While among infants at 28^+0^–31^+6^ weeks, mothers of 1,219/1,539 (79.2%) neonates were diagnosed with preeclampsia/eclampsia.

### Maternal and infant characteristics

The maternal and infant characteristics of HDP and non-HDP groups are presented in Table 2. Compared with the non-HDP group, mothers in the HDP group were significantly older. Mothers with HDP were more likely to be primigravida, to receive antenatal steroids and MgSO_4_, and to deliver *via* cesarean section, but were less likely to have prolonged rupture of membrane (PROM) >24 h and multiple births. Infants in the HDP group had larger gestational ages but lower birth weights than infants in the non-HDP group. Infants born to mothers with HDP were more likely to be SGA, female, and inborn.

**Table 2:** Maternal and VPIs characteristics born to non-HDP and HDP mothers.

Characteristics, *n*/*N* (%)	Non-HDP (*N* = 7518)	HDP (*N* = 1744)	*P* value
Maternal age, years, mean (SD)	30.5 (4.9)	32.1 (5.1)	<0.01
Primigravida	3,755/7,470 (50.3)	917/1,732 (52.9)	0.04
Prenatal care	7,209/7,281 (99.0)	1,681/1,697 (99.1)	0.86
Maternal diabetes	1,282/7,499 (17.1)	296/1,723 (17.2)	0.93
PROM >24 h	2,069/7,091 (29.2)	77/1,666 (4.6)	<0.01
Antenatal steroids	5,121/6,849 (74.8)	1,300/1,613 (80.6)	<0.01
MgSO_4_	2,779/6,307 (44.1)	1,035/1,476 (70.1)	<0.01
Cesarean section	3,527/7,489 (47.1)	1,569/1,738 (90.3)	<0.01
Multiple birth	2,466/7,518 (32.8)	289/1,744 (16.6)	<0.01
Gestational age, weeks, median (IQR)	29.7 (28.3–31.0)	30.3 (29.1-31.1)	<0.01
Birth weight, Grams, mean (SD)	1,355.4 (318.3)	1,188.4 (287.1)	<0.01
LGA, >90th percentile	252/7,518 (3.4)	21/1,744 (1.2)	<0.01
SGA, <10th percentile	218/7,518 (2.9)	419/1,744 (24.0)	<0.01
Female	3,170/7,511 (42.2)	844/1,740 (48.5)	<0.01
Apgar score at 5 min <7	513/7,067 (7.3)	126/1,658 (7.6)	0.63
Inborn	4,789/7,518 (63.7)	1,226/1,744 (70.3)	<0.01

HDP, hypertensive disorder of pregnancy; VPI, very preterm infants; SGA, small for gestational age.

^a^ Denominators for some rates were not equal to the total number of infants in certain group due to missing data.

### Neonatal outcomes of VPIs born to HDP and non-HDP mothers

Though, infants in the HDP group showed higher observed rates of mortality or any morbidity than infants in the non-HDP group (50.2% vs. 47.2%, crude OR 1.13, 95% CI 1.02–1.26), there were no significant differences of morality or any morbidity between the two groups after adjustment. Similar results applied to sepsis and BPD. Other outcomes did not differ between HDP and non-HDP groups both before and after adjustment (Table 3).

**Table 3:** Neonatal outcomes of very preterm infants <32 weeks born to non-HDP and HDP mothers.

Outcomes, *n*/*N* (%)	Non-HDP^a^ (*N* = 7,518)	HDP (*N* = 1,744)	Crude OR (95% CI)^b^	Model 1 adjusted OR^a,b^ (95% CI)	Model 2 adjusted OR^a,c^ (95% CI)
Mortality or any morbidity	3,546/7,518 (47.2)	876/1,744 (50.2)	1.13 (1.02–1.26)	0.99 (0.88–1.12)	0.95 (0.83–1.09)
Overall death	890/7,518 (11.8)	212/1,744 (12.2)	1.03 (0.88–1.21)	1.04 (0.86–1.27)	1.15 (0.91–1.47)
Early death (<7 days)	498/7,509 (6.6)	127/1,741 (7.3)	1.11 (0.91–1.36)	1.07 (0.80–1.41)	1.20 (0.89–1.62)
Sepsis	665/7,189 (9.3)	185/1,671 (11.1)	1.22 (1.03–1.45)	1.02 (0.84–1.24)	0.99 (0.78–1.25)
Early-onset sepsis	117/7,518 (1.6)	12/1,744 (0.7)	0.44 (0.24–0.80)	0.47 (0.17–1.30)	0.49 (0.18–1.32)
Late-onset sepsis	563/7,178 (7.8)	175/1,669 (10.5)	1.38 (1.15–1.65)	1.11 (0.92–1.34)	1.08 (0.86–1.35)
NEC, stage ≥2	387/7,518 (5.2)	98/1,744 (5.6)	1.10 (0.87–1.38)	0.86 (0.64–1.15)	0.74 (0.53–1.05)
Severe ROP, stage ≥3^d^	262/5,799 (4.5)	48/1,359 (3.5)	0.77 (0.57–1.06)	0.78 (0.49–1.26)	0.88 (0.46–1.71)
Severe brain injury, IVH grade ≥3 and/or cystic PVL^e^	742/6,344 (11.7)	159/1,477 (10.8)	0.91 (0.76–1.09)	0.93 (0.76–1.13)	0.85 (0.65–1.10)
BPD at corrected GA 36 weeks or at discharge	2,602/7,502 (34.7)	647/1,731 (37.4)	1.12 (1.01–1.25)	1.01 (0.89–1.15)	1.00 (0.86–1.15)
BPD at corrected GA 36 weeks	1,464/3,915 (37.4)	394/1,113 (35.4)	0.92 (0.80–1.05)	1.05 (0.89–1.23)	1.00 (0.83–1.20)

HDP, hypertensive disorder of pregnancy; NEC, necrotizing enterocolitis; ROP, retinopathy of prematurity; IVH, intraventricular hemorrhage; PVL, periventricular leukomalacia; GA, gestational age.

^a^ Non-HDP as reference group.

^b^ Model 1: Adjusted for maternal age, primigravida, maternal diabetes, gestational age, birth weight, sex, and multiple birth.

^c^ Model 2: Adjusted for the same covariates as in model 1 and prenatal care, antenatal corticosteroids, cesarean section, MgSO_4_, and inborn.

^d^ ROP was calculated among infants with ROP screening results.

^e|^ Severe brain impairment was calculated among infants with certain neuroimaging results.

For infants at 24^+0^–27^+6^ weeks, no difference in crude rates of neonatal outcomes was observed between HDP and non-HDP groups. With the adjustment, however, HDP has an independently significant association with decreased odds of NEC [adjusted OR (aOR) 0.35, 95% CI 0.14–0.87 in model 2] and severe ROP (aOR 0.39, 95% CI 0.17–0.88 in model 1, aOR 0.27, 95% CI 0.09–0.83 in model 2) but increased odds of severe brain injury (aOR 1.67, 95% CI 1.04–2.67 in model 1) (Supplementary Table S1). No significant association between HDP and any neonatal outcomes was identified among infants at 28^+0^–31^+6^ weeks (Supplementary Table S2).

### Neonatal outcomes and preeclampsia/eclampsia

Among 1,744 infants born to mothers with HDP, 1,688 infants had information on the diagnosis of preeclampsia/eclampsia of their mothers (Table 1). The baseline characteristics of infants born to mothers with (78.4%, 1,324/1,688) or without (21.6%, 364/1,688) preeclampsia/eclampsia were shown in Supplementary Table S3. The overall rates of mortality or any morbidity were 49.1% among infants born to mothers with preeclampsia/eclampsia and 53.0% among infants born to mothers without preeclampsia/eclampsia (crude OR 0.86, 95% CI 0.68–1.08) (Table 4). In multivariate regression analysis, infants born to mothers with preeclampsia/eclampsia had significantly lower odds of early death (aOR 0.56, 95% CI 0.34–0.90 in model 1) and severe ROP (aOR 0.28, 95% CI 0.12–0.66 in model 2) (Table 4).

**Table 4:** Comparison of neonatal outcomes between very preterm infants born to mothers with or without preeclampsia/eclampsia.

Outcomes, *n*/*N* (%)	No pre- and/or eclampsia^a^ (*N* = 364)	Pre- and/or eclampsia (*N* = 1,324)	Crude OR (95% CI)^a^	Model 1 adjusted OR^a,b^ (95% CI)	Model 2 adjusted OR^a,c^ (95% CI)
Mortality or any morbidity	193/364 (53.0)	650/1,324 (49.1)	0.86 (0.68–1.08)	0.94 (0.72–1.22)	0.93 (0.71–1.22)
Overall death	50/364 (13.7)	152/1,324 (11.5)	0.81 (0.58–1.15)	0.81 (0.55–1.20)	0.98 (0.58–1.66)
Early death (<7 days)	35/363 (9.6)	86/1,322 (6.5)	0.65 (0.43–0.98)	0.56 (0.34–0.90)	0.69 (0.36–1.33)
Sepsis	33/341 (9.7)	147/1,277 (11.5)	1.21 (0.82–1.81)	1.26 (0.88–1.79)	1.21 (0.76–1.95)
Early-onset sepsis	5/364 (1.4)	7/1,324 (0.5)	0.38 (0.12–1.21)	0.44 (0.15–1.31)	0.37 (0.09–1.50)
Late-onset sepsis	29/340 (8.5)	141/1,276 (11.1)	1.33 (0.88–2.03)	1.38 (0.94–2.02)	1.38 (0.80–2.36)
NEC, stage ≥2	20/364 (5.5)	76/1,324 (5.7)	1.05 (0.63–1.74)	1.16 (0.71–1.89)	0.91 (0.45–1.84)
Severe ROP, stage ≥3^d^	15/270 (5.6)	30/1,050 (2.9)	0.50 (0.27–0.94)	0.54 (0.27–1.06)	0.28 (0.12–0.66)
Severe brain injury, IVH grade ≥3 and/or cystic PVL^e^	36/298 (12.1)	113/1,134 (10.0)	0.81 (0.54–1.20)	0.95 (0.62–1.44)	1.00 (0.60–1.66)
BPD at corrected GA 36 weeks or at discharge	150/358 (41.9)	471/1,317 (35.8)	0.77 (0.61–0.98)	0.82 (0.62–1.07)	0.79 (0.58–1.06)

NEC, necrotizing enterocolitis; ROP, retinopathy of prematurity; IVH, intraventricular hemorrhage; PVL, periventricular leukomalacia; GA, gestational age.

^a^ No preeclampsia and/or eclampsia as the reference group.

^b^ Model 1: Adjusted for maternal age, primigravida, maternal diabetes, gestational age, birth weight, sex, and multiple birth.

^c^ Model 2: Adjusted for the same covariates as in model 1 and prenatal care, antenatal corticosteroids, cesarean section, MgSO_4_, and inborn.

^d^ ROP was calculated among infants with ROP screening results.

^e^ Severe brain impairment was calculated among infants with certain neuroimaging results.

**Table 5:** Comparison of neonatal outcomes among very preterm infants born to mothers without HDP, mothers with preeclampsia/eclampsia, and mothers with HDP but no preeclampsia/eclampsia.

	Pre- and/or eclampsia vs. Non-HDP			HDP but no pre- and/or eclampsia vs. Non-HDP		
Outcomes, *n*/*N* (%)	Crude OR (95% CI)^a^	Model 1 Adjusted OR^a,b^ (95% CI)	Model 2 Adjusted OR^a,c^ (95% CI)	Crude OR (95% CI)^a^	Model 1 adjusted OR^a,b^ (95% CI)	Model 2 adjusted OR^a,c^ (95% CI)
Mortality or any morbidity	1.27 (1.02–1.56)	1.06 (0.86–1.31)	1.02 (0.81–1.30)	1.08 (0.96–1.21)	0.97 (0.85–1.11)	0.91 (0.79–1.05)
Overall death	1.19 (0.87–1.61)	1.13 (0.82–1.54)	1.23 (0.90–1.68)	0.97 (0.80–1.16)	0.96 (0.75–1.21)	1.08 (0.80–1.47)
Early death (<7 days)	1.50 (1.05–2.15)	1.39 (0.91–2.12)	1.51 (0.99–2.31)	0.98 (0.77–1.24)	0.89 (0.63–1.24)	1.01 (0.71–1.44)
Sepsis	1.05 (0.73–1.52)	0.90 (0.65–1.25)	0.94 (0.63–1.39)	1.28 (1.06–1.54)	1.07 (0.86–1.33)	1.01 (0.78–1.32)
Early-onset sepsis	0.88 (0.36–2.17)	1.13 (0.40–3.20)	1.30 (0.47–3.62)	0.34 (0.16–0.72)	0.31 (0.08–1.24)	0.26 (0.07–0.95)
Late-onset sepsis	1.10 (0.74–1.62)	0.89 (0.63–1.25)	0.90 (0.59–1.36)	1.46 (1.20–1.77)	1.18 (0.96–1.45)	1.16 (0.89–1.50)
NEC, stage ≥ 2	1.07 (0.68–1.70)	0.86 (0.56–1.32)	0.74 (0.41–1.33)	1.12 (0.87–1.45)	0.89 (0.64–1.23)	0.77 (0.52–1.13)
Severe ROP, stage ≥ 3^d^	1.24 (0.73–2.12)	1.21 (0.66–2.22)	1.43 (0.70–2.91)	0.62 (0.42–0.91)	0.67 (0.39–1.15)	0.74 (0.33–1.64)
Severe brain injury, IVH grade ≥ 3 and/or cystic PVL^e^	1.04 (0.73–1.48)	0.96 (0.69–1.35)	0.79 (0.50–1.25)	0.84 (0.68–1.03)	0.89 (0.73–1.09)	0.85 (0.66–1.10)
BPD at corrected GA 36 weeks or at discharge	1.36 (1.10–1.68)	1.18 (0.95–1.46)	1.18 (0.93–1.51)	1.05 (0.93–1.19)	0.96 (0.82–1.11)	0.92 (0.78–1.09)

HDP, hypertensive disorder of pregnancy; NEC, necrotizing enterocolitis; ROP, retinopathy of prematurity; IVH, intraventricular hemorrhage; PVL, periventricular leukomalacia; GA, gestational age.

^a^ Non-HDP as reference group.

^b^ Model 1: Adjusted for maternal age, primigravida, maternal diabetes, gestational age, birth weight, sex, and multiple birth.

^c^ Model 2: Adjusted for the same covariates as in model 1 and prenatal care, antenatal corticosteroids, cesarean section, MgSO_4_, and inborn.

^d^ ROP was calculated among infants with ROP screening results.

^e^ Severe brain impairment was calculated among infants with certain neuroimaging results.

## Discussion

We found a high HDP incidence of 18.8% among mothers of VPIs, and the rate increased significantly with increasing gestational age. In our entire cohort, the rate of maternal HDP is slightly higher than in previous studies in other countries and in China (3, 14, 23). We may overestimate the rate because CHNN participating sites are all tertiary hospitals with high national-ranking and high-level of medical treatment, so that our cohort is more likely to admit infants born to mothers with pregnancy complications including HDP. The changing of maternal characteristics may also play a role, for example, more women of advanced reproductive age and more multiple births due to the popularity of assisted reproductive technology may result in a higher rate of HDP (33). Our results, combined with the previous studies, emphasized the gradual rising of the incidence of HDP, which demonstrates that the research on HDP deserves significant attention.

Though we did not find any significant association between neonatal outcomes and HDP among overall VPIs, our study showed that infants had different demographic characteristics between HDP and non-HDP groups. Infants born to mothers with HDP had higher gestational age but lower birth weight, which expressed a higher rate of SGA or intrauterine growth restriction (IUGR). The pathophysiologic mechanisms of vasospasm and decreased intravascular volume may play an important role in fetal growth and may explain the increased incidence of IUGR in the HDP group (34, 35). Preeclampsia could overwhelm the metabolic adaptations and result in IUGR through another pathway by reducing organ perfusion and further compromising organ blood flow (36, 37). The lower rate of male infants in the HDP group was consistent with previous studies (38, 39). The reason should be that although male embryos could be the risk factor for preeclampsia, the preponderance of preterm birth was female (38, 40). Given that SGA infants and male infants tend to have worse neonatal outcomes, since we adjusted for gestational age, birth weight, and sex in the multivariate regression, the actual effect of HDP on neonatal outcomes might be eliminated to some degree.

Within the subgroup of extremely preterm infants (EPIs) at 24 ^+ 0^–27 ^+ 6^ weeks, HDP was associated with lower odds of NEC and severe ROP. Significantly higher rates of cesarean section, antenatal steroid administration, MgSO_4_ application, and a lower rate of PROM >24 h in the HDP group indicated that pregnancies affected by HDP are more well-planed, monitored, and treated (14). The result of decreased odds of NEC may happen by chance due to the overall low incidence of NEC in our cohort and the small case number in subgroup of EPIs. Reduced severe ROP in the HDP group may be partially attributed to the lower VEGF levels and higher levels of antiangiogenic factors in preeclampsia (20, 41), which was also confirmed by our subgroup analysis of preeclampsia/eclampsia within the HDP group. However, even though proportion of preeclampsia/eclampsia is larger in large gestational age at 28^+0^–31^+6^ weeks, a significant association between HDP and ROP was not found in this subgroup, which needs to be further investigated.

Higher odds of severe brain injury were also found significant among EPIs in our study. The influence of HDP, especially preeclampsia, on the neurodevelopmental disorders of offspring is gradually recognized. Numerous studies have shown that HDP had increased susceptibility to multiple neurodevelopmental disorders such as cognitive impairment, depression, schizophrenia, and an autism spectrum disorder (15, 42–45). HDP, as a state of maternal immune activation (MIA), may affect fetal neurodevelopment through inflammation (42, 46). Though HDP was reported to have a relationship with reduced IVH, the results were inconsistent across studies and countries (14, 16). Furthermore, future study with long-term follow-up is necessary to confirm the impact of HDP on post-neonatal neurodevelopmental outcomes.

Our study was based on the largest cohort of very preterm infants in China. Detailed data collection enables us to further discuss the impact of preeclampsia/eclampsia. However, other subtypes of HDP were not discussed separately in this study due to insufficient cases of chronic hypertension. Information about the variations in diagnosis and management of HDP among sites was not demonstrated. The control of maternal blood pressure was not recorded in our database. We only included infants admitted to NICUs instead of all live births, which may introduce selection bias. Our database did not collect data on other pregnancy complications, which might also be confounders.

In conclusion, nearly one-fifth of VPIs were born to mothers with HDP in Chinese NICUs, and mothers with HDP generally had better perinatal care. No significant association was identified between HDP and adverse neonatal short-term outcomes of VPIs, while long-term follow-up of theses infants is needed.

## Group information of the Chinese neonatal network

Chairmen: Shoo K. Lee, MBBS, Mount Sinai Hospital, University of Toronto; Chao Chen, MD, Children’s Hospital of Fudan University. Vice-Chairmen: Lizhong Du, MD, Children's Hospital of Zhejiang University School of Medicine; Wenhao Zhou, Children’s Hospital of Fudan University. Site principle investigators of the Chinese Neonatal Network: Children's Hospital of Fudan University: Yun Cao, MD; The Third Affiliated Hospital of Zhengzhou University: Falin Xu, MD; Tianjin Obstetrics & Gynecology Hospital: Xiuying Tian, MD; Guangzhou Women and Children’s Medical Center: Huayan Zhang, MD; Children’s Hospital of Shanxi: Yong Ji, MD; Northwest Women's and Children's Hospital: Zhankui Li, MD; Gansu Provincial Maternity and Child Care Hospital: Jingyun Shi, MD; Shengjing Hospital of China Medical University: Xindong Xue, MD; Shenzhen Maternity and Child Health Care Hospital: Chuanzhong Yang, MD; Quanzhou Women and Children’s Hospital: Dongmei Chen, MD; Suzhou Municipal Hospital affiliated to Nanjing Medical University: Sannan Wang, MD; Guizhou Women and Children’s Hospital/Guiyang Children’s Hospital: Ling Liu, MD; Hunan Children’s Hospital: Xirong Gao, MD; The First Bethune Hospital of Jilin University: Hui Wu, MD; Fujian Maternity and Child Health Hospital, Affiliated Hospital of Fujian Medical University: Changyi Yang, MD; Nanjing Maternity and Child Health Care Hospital: Shuping Han, MD; Qingdao Women and Children’s Hospital: Ruobing Shan, MD; The Affiliated Hospital of Qingdao University: Hong Jiang, MD; Children’s Hospital of Shanghai: Gang Qiu, MD; Women and Children's Hospital of Guangxi Zhuang Autonomous Region: Qiufen Wei, MD; Children’s Hospital of Nanjing Medical University: Rui Cheng, MD; Henan Children’s Hospital: Wenqing Kang, MD; The First Affiliated Hospital of Xinjiang Medical University: Mingxia Li, MD; Foshan Women and Children’s Hospital: Yiheng Dai, MD; The First Affiliated Hospital of Anhui Medical University: Lili Wang, MD; Shanghai First Maternity and Infant Hospital: Jiangqin Liu MD; Yuying Children's Hospital Affiliated to Wenzhou Medical University: Zhenlang Lin, MD; Children’s Hospital of Chongqing Medical University: Yuan Shi, MD; The First Affiliated Hospital of Zhengzhou University: Xiuyong Cheng, MD; The First Affiliated Hospital of USTC, Division of Life Sciences and Medicine, University of Science and Technology of China: Jiahua Pan, MD; Shaanxi Provincial People’s Hospital: Qin Zhang, MD; Children's Hospital of Soochow University: Xing Feng, MD; Wuxi Maternity and Child Healthcare Hospital: Qin Zhou, MD; People's Hospital of Xinjiang Uygur Autonomous Region: Long Li, MD; The Second Xiangya Hospital of Central South University: Pingyang Chen, MD; Qilu Children’s Hospital of Shandong University: Xiaoying Li, MD; Hainan Women and Children’s Hospital: Ling Yang, MD; Xiamen Children’s Hospital: Deyi Zhuang, MD; Xinhua Hospital affiliated to Shanghai Jiao Tong University School of Medicine: Yongjun Zhang, MD; Shanghai Children’s Medical Center, Shanghai Jiao Tong University School of Medicine: Jianhua Sun, MD; Shenzhen Children’s Hospital: Jinxing Feng, MD; Children's Hospital Affiliated to Capital Institute of Pediatrics: Li Li, MD; Women and Children’s Hospital, School of Medicine, Xiamen university: Xinzhu Lin, MD; General Hospital of Ningxia Medical University: Yinping Qiu, MD; First Affiliated Hospital of Kunming Medical University: Kun Liang, MD; Hebei Provincial Children's Hospital: Li Ma, MD; Jiangxi Provincial Children’s Hospital: Liping Chen, MD; Fuzhou Children’s Hospital of Fujian Province: Liyan Zhang, MD; First Affiliated Hospital of Xian Jiao Tong University: Hongxia Song, MD; Dehong people's Hospital of Yunnan Province: Zhaoqing Yin, MD; Beijing Children's Hospital, Capital Medical University: Mingyan Hei, MD; Zhuhai Center for Maternal and Child Health Care: Huiwen Huang, MD; Guangdong Women and Children's Hospital: Jie Yang, MD; Dalian Municipal Women and Children’s Medical Center: Dong Li, MD; Peking Union Medical College Hospital: Guofang Ding, MD; Obstetrics & Gynecology Hospital of Fudan University: Jimei Wang, MD; Shenzhen Hospital of Hongkong University: Qianshen Zhang, MD; Children's Hospital of Zhejiang University School of Medicine: Xiaolu Ma, MD

## Data Availability

The raw data supporting the conclusions of this article will be made available by the authors, without undue reservation.
