# Supplementary material for: Impact of hypertensive disorders of pregnancy on neonatal outcomes among infants born at 24+0–31+6 weeks’ gestation in China: A multicenter cohort study
Source: Front Pediatr. 2023 Feb 23;11:1005383. doi: 10.3389/fped.2023.1005383 (PMC9996092; doi:10.3389/fped.2023.1005383)
Supplement: Supplementary file 1 [file Table1.doc]

Supplement Table 1. Neonatal outcomes of very preterm infants 24^+0^ - 27^+6^ weeks born to non-HDP and HDP mothers

| Outcomes, n/N (%) | Non-HDP *  (N=1409) | HDP  (N=158) | Crude OR (95%CI) * | Model 1 Adjusted OR * † (95%CI) | Model 2 Adjusted OR * ‡ (95%CI) |
| --- | --- | --- | --- | --- | --- |
| Mortality or any morbidity | 1076/1409 (76.4) | 122/158 (77.2) | 1.05 (0.71, 1.55) | 0.98 (0.60, 1.60) | 1.12 (0.62, 2.00) |
| Overall death | 434/1409 (30.8) | 48/158 (30.4) | 0.98 (0.69, 1.40) | 1.07 (0.66, 1.73) | 1.05 (0.58, 1.90) |
| Early death (<7 days) | 249/1405 (17.7) | 27/158 (17.1) | 0.96 (0.62, 1.48) | 0.97 (0.54, 1.75) | 0.86 (0.42, 1.76) |
| Sepsis | 186/1241 (15.0) | 21/149 (14.1) | 0.93 (0.57, 1.52) | 0.97 (0.55, 1.70) | 0.93 (0.45, 1.93) |
| Early-onset sepsis | 39/1409 (2.8) | 3/158 (1.9) | 0.68 (0.21, 2.23) | 0.85 (0.24, 2.94) | 1.16 (0.31, 4.36) |
| Late-onset sepsis | 154/1235 (12.5) | 18/148 (12.2) | 0.97 (0.58, 1.64) | 0.95 (0.53, 1.70) | 0.83 (0.39, 1.76) |
| NEC, stage≥2 | 96/1409 (6.8) | 9/158 (5.7) | 0.83 (0.41, 1.67) | 0.76 (0.42, 1.38) | 0.35 (0.14, 0.87) |
| Severe ROP, stage≥3 § | 131/969 (13.5) | 9/106 (8.5) | 0.59 (0.29, 1.20) | 0.39 (0.17, 0.88) | 0.27 (0.09, 0.83) |
| Severe brain injury, IVH grade≥3 and/or cystic PVL || | 226/1102 (20.5) | 34/125 (27.2) | 1.45 (0.95, 2.21) | 1.67 (1.04, 2.67) | 1.48 (0.83, 2.65) |
| BPD at corrected GA 36 weeks or at discharge | 911/1405 (64.8) | 104/158 (65.8) | 1.04 (0.74, 1.48) | 0.99 (0.61, 1.63) | 1.20 (0.75, 1.90) |
| BPD at corrected GA 36 weeks | 437/773 (56.5) | 51/88 (58.0) | 1.06 (0.68, 1.66) | 1.12 (0.66, 1.89) | 1.12 (0.59, 2.12) |
| * Non-HDP as reference group. | | | | | |
| † Model 1: Adjust for maternal age, primigravida, maternal diabetes, gestational age, birth weight, sex, multiple birth.  ‡ Model 2: Adjust for the same covariates as in model 1 and prenatal care, antenatal corticosteroids, cesarean section, MgSO4, and inborn. | | | | | |
| § ROP was calculated among infants with ROP screening results.  || Severe brain impairment was calculated among infants with certain neuroimaging results. | | | | | |

Supplement Table 2. Neonatal outcomes of very preterm infants 28^+0^ - 31^+6^ weeks born to non-HDP and HDP mothers

| Outcomes, n/N (%) | Non-HDP *  (N=6109) | HDP  (N=1586) | Crude OR (95%CI) * | Model 1 Adjusted OR * † (95%CI) | Model 2 Adjusted OR * ‡ (95%CI) |
| --- | --- | --- | --- | --- | --- |
| Mortality or any morbidity | 2470/6109 (40.4) | 754/1586 (47.5) | 1.34 (1.20, 1.49) | 0.99 (0.88, 1.11) | 0.92 (0.81, 1.05) |
| Overall death | 456/6109 (7.5) | 164/1586 (10.3) | 1.43 (1.19, 1.73) | 1.00 (0.80, 1.25) | 1.15 (0.89, 1.50) |
| Early death (<7 days) | 249/6104 (4.1) | 100/1583 (6.3) | 1.59 (1.25, 2.01) | 1.04 (0.75, 1.43) | 1.32 (0.91, 1.90) |
| Sepsis | 479/5948 (8.1) | 164/1522 (10.8) | 1.38 (1.14, 1.66) | 1.06 (0.88, 1.28) | 1.04 (0.82, 1.32) |
| Early-onset sepsis | 78/6109 (1.3) | 9/1586 (0.6) | 0.44 (0.22, 0.88) | 0.37 (0.12, 1.16) | 0.32 (0.10, 1.00) |
| Late-onset sepsis | 409/5943 (6.9) | 157/1521 (10.3) | 1.56 (1.28, 1.89) | 1.18 (0.98, 1.43) | 1.18 (0.93, 1.50) |
| NEC, stage≥2 | 291/6109 (4.8) | 89/1586 (5.6) | 1.19 (0.93, 1.52) | 0.88 (0.63, 1.21) | 0.79 (0.54, 1.16) |
| Severe ROP, stage≥3 ^d^ | 131/4830 (2.7) | 39/1253 (3.1) | 1.15 (0.80, 1.66) | 0.93 (0.56, 1.55) | 1.18 (0.60, 2.34) |
| Severe brain injury, IVH grade≥3 and/or cystic PVL ^e^ | 516/5242 (9.8) | 125/1352 (9.3) | 0.93 (0.76, 1.15) | 0.83 (0.67, 1.02) | 0.77 (0.59, 1.02) |
| BPD at corrected GA 36 weeks or at discharge | 1691/6097 (27.7) | 543/1573 (34.5) | 1.37 (1.22, 1.55) | 1.00 (0.87, 1.14) | 0.95 (0.82, 1.10) |
| BPD at corrected GA 36 weeks | 1027/3142 (32.7) | 343/1025 (33.5) | 1.04 (0.89, 1.20) | 1.03 (0.87, 1.23) | 0.96 (0.79, 1.17) |
| * Non-HDP as reference group. | | | | | |
| † Model 1: Adjust for maternal age, primigravida, maternal diabetes, gestational age, birth weight, sex, multiple birth.  ‡ Model 2: Adjust for the same covariates as in model 1 and prenatal care, antenatal corticosteroids, cesarean section, MgSO4, and inborn. | | | | | |
| § ROP was calculated among infants with ROP screening results.  || Severe brain impairment was calculated among infants with certain neuroimaging results. | | | | | |

Supplement Table 3, Maternal and infant characteristics of very preterm infants born to born to mothers with or without preeclampsia/eclampsia

| Characteristics, n/N (%) | None preeclampsia/eclampsia  (N=364) | Preeclampsia/eclampsia  (N=1324) | P-value |
| --- | --- | --- | --- |
| Maternal age, years, mean (SD) | 32.2 (5.2) | 32.1 (5.1) | 0.84 |
| Primigravida | 202/362 (55.8) | 689/1314 (52.4) | 0.26 |
| Prenatal care | 347/349 (99.4) | 1281/1294 (99.0) | 0.45 |
| Maternal Diabetes | 80/362 (22.1) | 207/1310 (15.8) | <.01 |
| PROM>24h | 35/348 (10.1) | 35/1274 (2.8) | <.01 |
| Antenatal steroids | 257/346 (74.3) | 1019/1235 (82.5) | <.01 |
| MgSO4 | 156/310 (50.3) | 867/1142 (75.9) | <.01 |
| Cesarean section | 282/363 (77.7) | 1242/1319 (94.2) | <.01 |
| Multiple birth | 81/364 (22.3) | 201/1324 (15.2) | <.01 |
| Gestational age, weeks, median (IQR) | 30.0 (29.0-31.0) | 30.3 (29.1-31.1) | 0.03 |
| Birth weight, grams, mean (SD) | 1215.3 (299.4) | 1180.1 (279.5) | 0.04 |
| LGA, >90^th^ percentile | 7/364 (1.9) | 12/1324 (0.9) | 0.15 |
| SGA, <10^th^ percentile | 72/364 (19.8) | 335/1324 (25.3) | 0.03 |
| Female | 165/364 (45.3) | 651/1320 (49.3) | 0.18 |
| Apgar score at 5 min <7 | 35/338 (10.4) | 86/1275 (6.8) | 0.03 |
| Inborn | 244/364 (67.0) | 973/1324 (73.5) | 0.02 |
